# Cardiac Remodeling and Functional Alterations in Heart Failure With Preserved Ejection Fraction (HFpEF): An Echocardiographic Assessment at a Tertiary Hospital in Bangladesh

**DOI:** 10.7759/cureus.94831

**Published:** 2025-10-17

**Authors:** Mohammad Walidur Rahman, Manzoor Mahmood, Dipal Krishna Adhikary, Chaudhury Meshkat Ahmed, Md. Harisul Hoque, Tanjima Parvin, Sajal Krishna Banerjee, Md. Fakhrul Islam Khaled

**Affiliations:** 1 Cardiology, Bangladesh Medical University, Dhaka, BGD

**Keywords:** diastolic dysfunction, global longitudinal strain, hfpef, pulmonary hypertension, structural remodeling

## Abstract

Background

Heart failure with preserved ejection fraction (HFpEF) is a complex pathophysiological entity characterized by varying degrees of cardiac structural and functional changes. This study evaluated the prevalence and pattern of cardiac remodeling and functional alterations in an HFpEF cohort in Bangladesh.

Methods

This study was conducted among 50 hospitalized HFpEF patients from December 2017 to March 2019 at Bangladesh Medical University. Echocardiographic evaluations were performed to assess left ventricular (LV) structure, systolic and diastolic function, and right ventricular (RV) systolic function. The LV global longitudinal strain (GLS) was measured to detect subclinical systolic dysfunction.

Results

The mean (SD) age of the participants was 65 (10) years, and 30 (60%) were male. Most patients had multiple comorbidities and presented in a decompensated state. Despite a mean LV ejection fraction of 60.4% (6.0%), the majority of cases, 28 (68%), showed reduced GLS after excluding nine cases of permanent atrial fibrillation (AF). Structural alterations of the LV were observed in 26 (52%) patients. Concentric LV hypertrophy and concentric remodeling were present in 21 (42%) of the assessed patients. LV diastolic dysfunction was found in 47 (94%) cases; however, diastolic dysfunction grading could not be performed in nine (19%) cases due to permanent AF. Six (13%) patients had grade I diastolic dysfunction, while 32 (68%) had grade II to III dysfunction. After excluding cases with permanent AF and moderate mitral valve regurgitation, the left atrial volume index (LAVi) was increased in 32 (91%) patients. Pulmonary hypertension was observed in 22 (44%) cases, and RV systolic impairment in five (10%) cases.

Conclusions

All patients in this study exhibited evidence of either cardiac remodeling or diastolic dysfunction. LV remodeling was less common, whereas diastolic dysfunction and LAVi enlargement were more prevalent. However, large-scale epidemiological studies are needed for better characterization of the HFpEF cohort in Bangladesh.

## Introduction

Heart failure with preserved ejection fraction (HFpEF) is a heterogeneous clinical syndrome that presents greater challenges than heart failure with reduced ejection fraction. These challenges stem from the lack of a unifying definition across trials, varying degrees of cardiac structural and functional changes, associations with multiple comorbidities that influence its presentation and progression, and a long list of failed therapeutic interventions [1,2]. The primary cardiac pathology implicated in this diverse syndrome is thought to be left ventricular (LV) diastolic dysfunction with associated concentric remodeling. However, alterations in cardiac structure, function, and underlying hemodynamic changes differ among HFpEF phenotypes [2]. The characteristics of HFpEF patients may also vary across geographical regions. Therefore, this study aimed to assess the pattern of cardiac remodeling and functional changes in HFpEF patients in Bangladesh.

## Materials and methods

Study design, place, and duration

This cross-sectional study was conducted from December 2017 to March 2019 in the Department of Cardiology, Bangladesh Medical University in Dhaka, Bangladesh. The Department of Cardiology has 95 beds and includes both clinical and interventional divisions, equipped with all the facilities of a modern cardiac center.

Inclusion criteria and exclusion criteria

Inclusion criteria included adult patients (age >18 years) diagnosed with HFpEF. The diagnosis of HFpEF was established according to the 2016 Heart Failure Guidelines of the European Society of Cardiology [3].

Exclusion criteria included patients hospitalized primarily for acute myocardial infarction or acute coronary syndrome; patients with severe valvular heart disease, prosthetic valves, pacemakers, congenital heart disease, or a poor echocardiographic window.

Echocardiographic evaluation

Echocardiographic assessments were performed using the Vivid E9 system (GE Healthcare, Oslo, Norway). The acquired images were independently analyzed by two investigators who were blinded to the patients’ clinical information.

LV dimensions were measured using M-mode, and LV ejection fraction (LVEF) was determined using the modified biplane Simpson’s method. LV mass was calculated by the area-length method and indexed to body surface area. LV hypertrophy and geometric alterations were categorized according to the recommendations of the American Society of Echocardiography and the European Association of Cardiovascular Imaging (ASE/EACVI) 2015 guidelines [4].

LV diastolic function was assessed according to the ASE/EACVI 2016 recommendations [5]. The left atrial volume index (LAVi) was measured using the biplane area-length method [4]. Right ventricular (RV) systolic function was evaluated using tricuspid annular plane systolic excursion (TAPSE) and tricuspid lateral annular systolic velocity (S′).

The presence and grading of valvular heart disease were determined using color Doppler, as well as image-guided pulsed and continuous-wave Doppler techniques, in accordance with the 2014 practice guidelines of the American College of Cardiology and the American Heart Association for the management of valvular heart disease [6].

**Figure 1:**
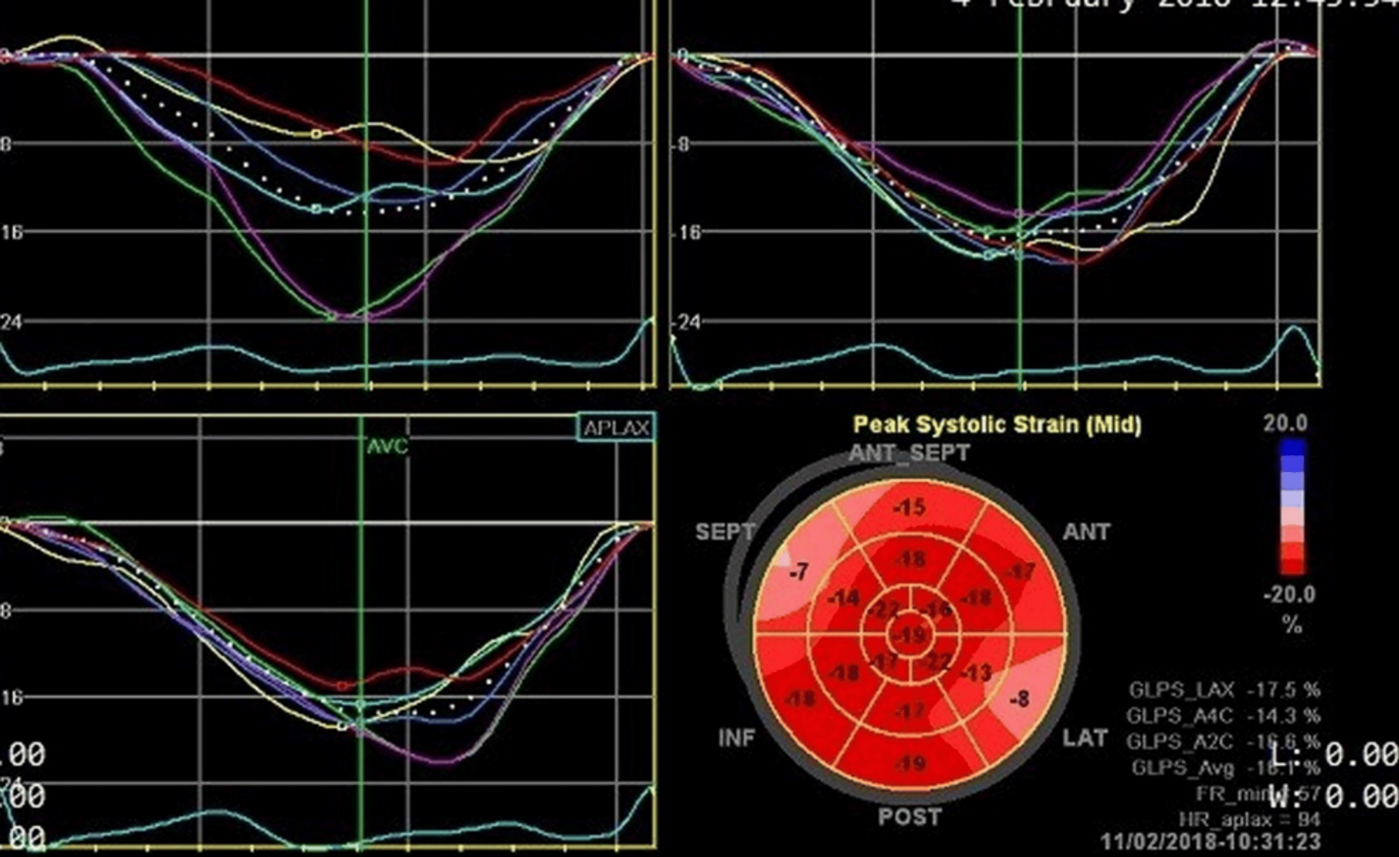
Measurement of LV GLS Speckle-tracking echocardiography was used to analyze LV GLS at frame rates greater than 50 s⁻¹. The peak longitudinal strains from each of the 17 LV segments, whether negative or positive, were automatically averaged from the apical four-chamber, two-chamber, and long-axis views to calculate LV GLS. GLS = global longitudinal strain; LV = left ventricular

Subclinical LV systolic dysfunction was evaluated by tissue Doppler-derived LV global longitudinal strain (GLS) (Figure 1). Speckle-tracking echocardiography was used to analyze LV longitudinal strains at frame rates >50 s⁻¹. The peak longitudinal strains from each of the 17 LV segments, whether negative or positive, were automatically averaged from the apical four-chamber, two-chamber, and long-axis views to calculate LV GLS.

Statistical analysis

All analyses were performed using Microsoft Excel 2019 (Microsoft Corporation, Redmond, WA, USA). Continuous data were expressed as mean (SD) and range, while categorical data were presented as frequencies and percentages.

## Results

Baseline characteristics

The mean (SD) age of the study population was 65 (10) years. Most participants were male, 30 (60%), and had multiple comorbidities and risk factors. Among these, hypertension (HTN) was the most common, present in 40 (80%) of the cases. Dyslipidemia was observed in 32 (64%) cases, and diabetes mellitus in 27 (54%). Coronary artery disease was found in 18 (36%) patients, and chronic kidney disease (CKD) in 15 (30%). Atrial fibrillation (AF) was present in 13 (26%) patients, of whom nine (18%) had permanent AF and four (8%) had paroxysmal AF. Overall, 31 (62%) of the study population were overweight or obese. The baseline characteristics of the study population are presented in Table 1.

**Table 1:** Baseline characteristics of the study population AF = atrial fibrillation; CAD = coronary artery disease; CKD = chronic kidney disease; DM = diabetes mellitus; HTN = hypertension

Characteristics	Value
Demographics	
Age, years (mean ± SD)	65 (10.1)
Male, n (%)	30 (60)
BMI >25, n (%)	31 (62)
Risk factors and comorbidities	
HTN, n (%)	40 (80)
Dyslipidemia, n (%)	32 (64)
DM, n (%)	27 (54)
CAD, n (%)	18 (36)
CKD, n (%)	15 (30)
AF, n (%)	13 (26)
Smoker, n (%)	12 (24)
Stroke, n (%)	3 (6)

Echocardiographic findings of the study population

All HFpEF patients in the study demonstrated either structural or functional alterations of the left ventricle according to the diagnostic criteria. Table 2 presents the echocardiographic findings of the study population.

**Table 2:** Echocardiographic findings of the study population A = peak late diastolic filling velocity during atrial contraction; e′ = mitral annular tissue velocity during early filling; E = peak early diastolic filling velocity; GLS = global longitudinal strain; IVRT = isovolumic relaxation time; LAVi = left atrial volume index; LV = left ventricle; RV = right ventricle; RVSP = right ventricular systolic pressure; TAPSE = tricuspid annular plane systolic excursion; TR Vmax = tricuspid regurgitation maximum velocity

Parameter	Mean (SD)	Range
LV structure		
End-diastolic dimension, cm	4.8 (0.5)	3.8-5.8
End-diastolic volume, mL/m²	49.4 (18.2)	20.6-89.6
End-systolic dimension, cm	3.2 (0.4)	2.3-4.3
End-systolic volume, mL/m²	20.0 (8.6)	8-43.8
Relative wall thickness, cm	0.38 (0.09)	0.23-0.50
Mass, g/m²	82.8 (25.9)	46.5-137.7
LV systolic function		
Fractional shortening, %	32.3 (4.8)	25-41
Ejection fraction, %	60.4 (6.0)	51-72
GLS, %	-16.5 (3.6)	-23.1 to -10
LV diastolic function		
E/A ratio	1.16 (0.7)	0.4-3.6
e′ lateral annulus, cm/s	6.2 (1.6)	3-9
e′ septal annulus, cm/s	5.1 (1.4)	2-7
E/e′ average	15.4 (4.6)	7.2-30.8
E/e′ septal	14.4 (3.2)	11.2-19
LAVi, mL/m²	41.4 (6.4)	30.6-61.1
TR Vmax, m/s	2.8 (0.5)	1.5-3.7
E deceleration time, ms	159.4 (49.5)	70-309
IVRT, ms	55.1 (6.3)	47.6-62
RV function		
TAPSE, cm	1.7 (0.3)	0.9-2.4
Tricuspid lateral annular systolic velocity (S′), cm/s	12.3 (2.7)	8.3-18
RVSP, mmHg	39.3 (13.3)	14-67

LV structural alteration

**Figure 2:**
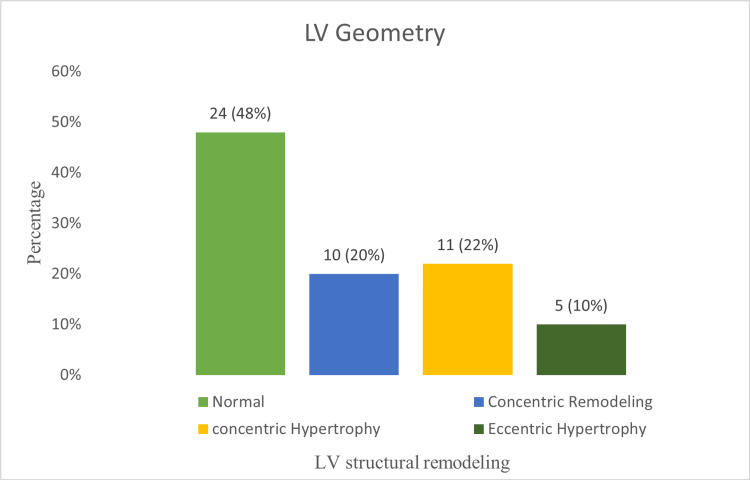
LV structural remodeling in HFpEF (n = 50) The x-axis represents LV structural remodeling, while the y-axis indicates the percentage of patients. HFpEF = heart failure with preserved ejection fraction; LV = left ventricular

The mean (SD) LV end-diastolic diameter was 48 (5) mm, and the mean LV end-diastolic volume was 49.4 (18.2) mL/m², both within the normal range (Table 2). However, the majority of patients, 26 (52%), showed structural alterations in the form of ventricular hypertrophy or concentric remodeling. Among them, 11 (22%) had concentric LV hypertrophy, five (10%) had eccentric hypertrophy, and 10 (20%) had concentric remodeling based on relative wall thickness (Figure 2).

LV systolic function

The mean (SD) LVEF was 60.4% (6.0%) (Table 2), and none of the participants had an LVEF below 50%. LV GLS could not be measured in nine cases with permanent AF. Among the 41 cases in which LV GLS was measurable, the mean LV GLS was −16.5% (3.6%) (Table 2), and it was reduced in 28 (68%) of these patients. Regional wall motion abnormalities were observed in 12 (24%) cases.

LV diastolic function

The LV diastolic function was normal in three (6%) cases. After excluding 15 cases with AF and moderate mitral regurgitation, only three (9%) patients had a normal LAVi. LAVi was mildly abnormal in 14 (40%) cases, moderately abnormal in another 14 (40%), and severely abnormal in four (11%). An increased left atrial (LA) diameter was noted in 27 (77%) cases.

Impairment in either lateral e′ {mean 6.2 (1.6) cm/s} or septal e′ {mean 5.1 (1.4) cm/s} was observed in 47 (94%) patients. Among those with AF, nine (69%) showed elevated average or septal E/e′ ratios, indicating high LV filling pressures. Diastolic dysfunction was present in 47 (94%) cases; grading could not be performed in nine (19%) due to permanent AF. Nevertheless, diastolic dysfunction was confirmed in all nine permanent AF cases, as evidenced by a shortened isovolumic relaxation time (IVRT) of 55.1 (6.3) ms and a septal E/e′ ≥ 11.

**Figure 3:**
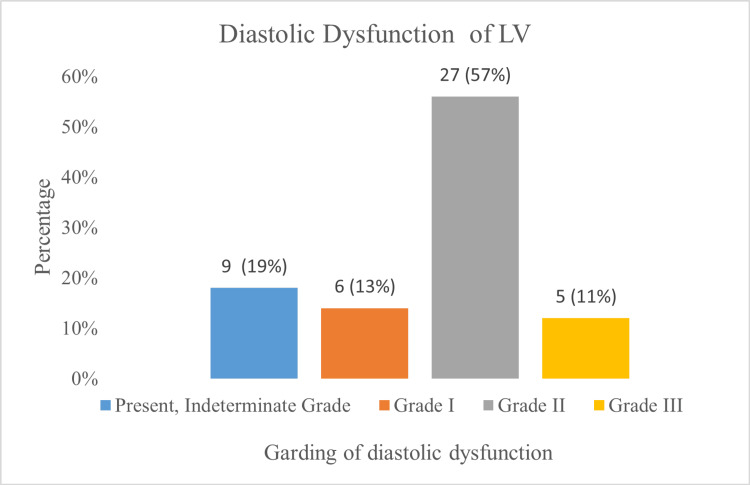
Grading of diastolic dysfunction in HFpEF (n = 47) The x-axis represents diastolic dysfunction grades, while the y-axis indicates the percentage of patients. HFpEF = heart failure with preserved ejection fraction; LV = left ventricular

Grade I diastolic dysfunction was found in six (13%) cases, grade II in 27 (57%), and grade III in five (11%). Figure 3 illustrates the distribution of diastolic dysfunction grades among HFpEF patients.

Right ventricle and pulmonary vasculature

Five (10%) cases showed RV systolic dysfunction, as indicated by reduced TAPSE and tissue Doppler-derived tricuspid lateral annular systolic velocity (S′). The tricuspid regurgitation (TR) jet was measurable in 39 (78%) cases, and a peak velocity greater than 2.8 m/s was present in 22 (44%) patients. The mean (SD) TR jet velocity was 2.8 (0.5) m/s. The mean (SD) pulmonary artery systolic pressure (PASP) was 39.3 (13.3) mmHg, and 22 (44%) patients had a PASP greater than 40 mmHg.

## Discussion

The baseline characteristics and structural and functional changes reported across HFpEF trials vary because of inconsistencies in diagnostic criteria and the inherent heterogeneity of the syndrome.

In contrast to the I-PRESERVE database, our patients were younger {65 (10) vs. 72 (7) years} and predominantly male (60% vs. 40%) [7]. In the JCARE-CARD registry, males accounted for 52.7% of patients in the HFpEF arm [8]. The male predominance in our study may be attributed to the lack of health-seeking behavior among females in Bangladesh. Rahman et al. [9] similarly reported that 75% of all heart failure patients in a tertiary-level hospital in Bangladesh were male. Most participants in our cohort had multiple comorbidities, with HTN being the most common (80%), comparable to other studies [10,11].

All patients in our study had either structural or functional LV abnormalities, or both, unlike the echocardiographic substudy of the TOPCAT trial, where 7% of subjects had normal LV geometry and LA size [12].

Structural remodeling

LV structural remodeling is defined by changes in LV mass, volume, and geometry. In our cohort, 52% of patients exhibited structural remodeling in the form of LV hypertrophy or concentric remodeling, closely resembling the I-PRESERVE substudy (54%) [7]. In PARAGON-HF, LV hypertrophy was present in 21% and concentric remodeling in 25% of patients [13]. Conversely, 86% of TOPCAT participants had altered LV geometry (concentric hypertrophy 43%, concentric remodeling 34%, and eccentric hypertrophy 9%) [12].

This discrepancy may be explained by the lower mean age (65.0 (10.1) vs. 70 (10) years), the lower prevalence of HTN (80% vs. 91%), and the higher prevalence of moderate to severe valvular lesions (10% vs. 14.4%) in our cohort compared to TOPCAT [12]. Although concentric hypertrophy is the expected finding in HFpEF, 10% of our participants demonstrated eccentric hypertrophy, consistent with the TOPCAT data, underscoring the heterogeneity of HFpEF [12].

LV systolic function

LV systolic function was assessed using the modified biplane Simpson’s method to measure LVEF, while subclinical dysfunction was evaluated using LV GLS, one of the strengths of this study. None of our participants showed overt LV systolic dysfunction, as indicated by a mean LVEF of 60.4% (6.0%). Similarly, a systematic review and meta-analysis by Jin et al. reported preserved systolic function in acute HFpEF patients, with a mean LVEF of 60.1% [14]. However, in the TOPCAT study, 13% of participants had an LVEF <50% [12].

After excluding nine cases of permanent AF, LV GLS was found to be significantly reduced in 68% of patients, despite preserved LVEF. This aligns with previous findings showing a high prevalence (66.7%) of abnormal GLS in HFpEF [15].

LV diastolic dysfunction

LV diastolic function was evaluated following the ASE/EACVI 2016 recommendations [5]. Diastolic dysfunction was present in 94% of cases, although grading could not be determined in 19% due to permanent AF. Grade I dysfunction was observed in 13% of cases, while grade II-III dysfunction was found in 68%. In contrast, diastolic dysfunction was absent in 31% of I-PRESERVE cases and 34% of TOPCAT cases [7,12].

Our inclusion of hospitalized patients with stricter diagnostic criteria likely accounts for the higher prevalence of diastolic dysfunction, as they were more decompensated than those in the I-PRESERVE cohort (New York Heart Association class IV: 74% vs. 3%) [10]. Compared to I-PRESERVE, our patients also had a higher prevalence of increased LAVi (91% vs. 66%) [10], possibly due to elevated LV end-diastolic pressures, present in 70% of cases.

Pulmonary HTN and RV systolic function

The prevalence of pulmonary HTN in our study (44%) was similar to that in the TOPCAT trial (36%) [12]. Pulmonary HTN in HFpEF is a recognized adverse prognostic factor associated with increased mortality and HF-related hospitalizations [16]. RV systolic dysfunction was observed in 10% of our patients, compared to 4% in TOPCAT [12].

Limitations

This study may not fully represent the broader Bangladeshi HFpEF population, as it included only hospitalized patients from a tertiary-level center, which may limit generalizability. Furthermore, variability in HFpEF diagnostic criteria across studies makes direct comparison with other trials challenging.

## Conclusions

Compared with Western HFpEF cohorts, our patients were younger, predominantly male, had multiple comorbidities, and were more often in a decompensated state. All patients exhibited structural or functional LV alterations, and LAVi was significantly higher than in other HFpEF trials. Despite preserved LVEF, subclinical LV systolic dysfunction detected by GLS was common, and diastolic dysfunction was more prevalent, likely reflecting the stricter inclusion criteria of this study.
